# gep2pep: a bioconductor package for the creation and analysis of pathway-based expression profiles

**DOI:** 10.1093/bioinformatics/btz803

**Published:** 2019-10-24

**Authors:** Farancesco Napolitano, Diego Carrella, Xin Gao, Diego di Bernardo

**Affiliations:** 1 Telethon Institute of Genetics and Medicine (TIGEM), Pozzuoli, NA 80078, Italy; 2 Computational Bioscience Research Center (CBRC), Computer, Electrical and Mathematical Sciences and Engineering (CEMSE), King Abdullah University of Science and Technology (KAUST), Thuwal 23955-6900, Kingdom of Saudi Arabia

## Abstract

**Summary:**

Pathway-based expression profiles allow for high-level interpretation of transcriptomic data and systematic comparison of dysregulated cellular programs. We have previously demonstrated the efficacy of pathway-based approaches with two different applications: the drug set enrichment analysis and the Gene2drug analysis. Here, we present a software tool that allows to easily convert gene-based profiles to pathway-based profiles and analyze them within the popular R framework. We also provide pre-computed profiles derived from the original Connectivity Map and its next generation release, i.e. the LINCS database.

**Availability and implementation:**

The tool is implemented as the R/Bioconductor package *gep2pep* and can be freely downloaded from https://bioconductor.org/packages/gep2pep.

**Supplementary information:**

Supplementary data are available at *Bioinformatics* online.

## 1 Introduction

The use of genome-wide expression profiling technologies has transformed the way in which scientists approach the study of molecular mechanisms. Millions of assays have been performed and public databases have been developed to collect such immense amount of data, thus improving both reproducibility of the original studies and reuseability for novel investigations (Rung and Brazma, 2013). Many different computational approaches have been developed to deal with the inherent complexity of transcriptomic data and to help mining biological knowledge out of them. The gene ontology (GO) (Ashburner *et al.*, 2000) has been one of the most popular tools to provide systematic insights into the activity of transcriptional programs by factoring in the expression of multiple genes together through the gene set enrichment analysis (GSEA).

GSEA is commonly used to aid biological interpretation downstream of transcriptomic data analyses. However, we recently proposed a different approach, in which GSEA is rather part of the data preprocessing phase. In particular, we use GSEA to convert gene expression profiles (GEPs) to pathway expression profiles (PEPs). This allows to develop analytic approaches that use dysregulated gene sets (although we refer to them as *pathways* for simplicity) as their elementary variables, as opposed to single genes. We demonstrated the efficacy of the approach with two different tools: (i) the drug set enrichment analysis (DSEA) (Napolitano *et al.*, 2016), which allows to identify pathways that are consistently dysregulated across a set of drugs, and (ii) the Gene2drug analysis (Napolitano *et al.*, 2018), which allows to perform gene-drug prioritization based on the pathways that the molecular target of interest is involved in. Both tools have been previously released as closed source web applications.

Here, we present *gep2pep*, an R/Bioconductor package that implements the pathway-based expression profiles paradigm. It supports conversion of large collections of GEPs to PEPs and provides routines to perform DSEA-like and Gene2drug-like analyses. Together with the package, we provide two large collections of PEPs, respectively derived from the Connectivity Map 2.0 (Cmap) (Lamb *et al.*, 2006) and its next generation released within the LINCS project (Subramanian *et al.*, 2017). Finally, we present an update of the DSEA tool which takes advantage of these new data (see Supplementary Material).

## 2 Materials and methods

### 2.1 Implementation

The *gep2pep* R/Bioconductor package supports the management of large collections of heterogeneous profiles, exploiting the HDF5 format in combination with the *Repo* (Napolitano, 2017) package for objects management. It also supports import of gene set collections from the *MSigDB* database (Liberzon *et al.*, 2011). Large datasets are handled through parallelization and partial results management support.

### 2.2 Converting GEPs to PEPs

PEPs are created from GEPs using GSEA. Given *N* GEPs related to a set of experimental *conditions*, and a database of *M* pathways such as those included in the GO, a GSEA is performed for each (*c*, *p*) pair, where *c* is a condition and *p* is a pathway. Therefore, each (*c*, *p*) pair is assigned an Enrichment Score (ES) and its corresponding *P*-value according to the Kolmogorov–Smirnov test (KST), giving rise to an *M* × *N* matrix *E* of ESs and an *M* × *N* matrix *P* of *P*-values. *gep2pep* allows to perform further PEP-based analyses using any of *E*, *P* or their element-wise product, according to: −*E* · log(*P*). *gep2pep* also implements merging of multiple PEPs into a single PEP, along the lines of Iorio *et al.* (2010) for GEPs. Given a collection of PEPs (*E*, *P*), the resulting merge is the row-wise average of *E*, and the row-wise aggregation of *P* by the Fisher’s method.

### 2.3 Analyzing PEPs

**Fig. 1:**
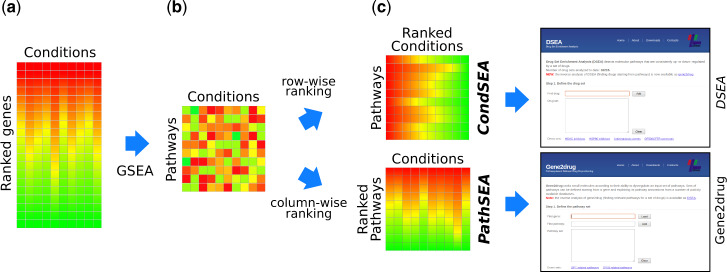
Pathway-based profiles creation and analysis using *gep2pep*. (**a**) A collection of ranked GEPs. (**b**) Using GSEA and a database of pathway annotations, enrichment of each pathway in each condition is computed, yielding PEPs. (**c**) Top: row-wise ranked PEPs are used by CondSEA to identify pathways that are consistently dysregulated for a set of conditions (used in DSEA). Bottom: column-wise ranked PEPs are used by PathSEA to identify conditions for which a set of pathways is consistently dysregulated (used in Gene2drug)

The *gep2pep* package supports two kinds of pathway-based analyses (see Fig. 1): condition set enrichment analysis (*CondSEA*) and pathway set enrichment analysis (*PathSEA*). Both are based on a GSEA-like procedure performed on PEPs as opposed to GEPs. CondSEA first ranks the PEPs row-wise. Then, given a set of conditions as input, it computes their KST within each row. Enriched rows correspond to pathways that are consistently dysregulated across the input conditions. When conditions are drug-induced GEPs, we call this approach *DSEA* (Napolitano *et al.*, 2016). Conversely, PathSEA first ranks the PEPs column-wise. Then, given a set of pathways as input, it computes their KST against each column. Enriched columns correspond to conditions in which most of the input pathways appear consistently dysregulated. When conditions are drug-induced GEPs and the input pathways are related to a pharmacological target, we call this approach *Gene2drug* (Napolitano *et al.*, 2018).

### 2.4 Pre-computed PEPs and the new DSEA web tool

Together with the package, here we release two large collections of pre-computed PEPs: one derived from the Cmap (Lamb *et al.*, 2006), including 1309 drug-induced profiles, and another one derived from the LINCS project (Subramanian *et al.*, 2017), including 17 974 profiles. In both cases, we used all the gene sets collections included in the MSigDB v6.1, which amount to a total of 14 645 gene sets. In the first case, we merged all the profiles obtained with the same drug, according to the rationale described in Iorio *et al.* (2010). In the latter case, which includes assays performed on many more cell lines, we included both profiles merged across cell lines and profiles merged across different drug dosages only. Overall, we computed (1309 + 17 974) * 14 645 ≈ 350 000 000 of ES—*P*-value pairs using a computer cluster. We have used these data to build a new version of the DSEA website (http://dsea.tigem.it/lincs), where the full dataset in the *gep2pep* format can be obtained (see Supplementary Material).

## 3 Conclusion

We introduced *gep2pep*, an R/Bioconductor package implementing a pathway-based approach to the analysis of GEPs. We also provided a large collection of pre-computed PEPs. We hope that an offline, structured and carefully documented tool for generic pathway-based approaches will allow more researchers to develop original applications under this new paradigm.

## Funding

This work has been supported by Fondazione Telethon and Fondazione Veronesi.

*Conflict of Interest*: none declared.

## Supplementary Material

btz803_Supplementary_Data
