# Supplementary material for: gep2pep: a bioconductor package for the creation and analysis of pathway-based expression profiles
Source: Bioinformatics. 2019 Oct 24;36(6):1944–5. doi: 10.1093/bioinformatics/btz803 (PMC7703749; doi:10.1093/bioinformatics/btz803)
Supplement: btz803_Supplementary_Data [file btz803_supplementary_data.pdf]

gep2pep: a Bioconductor package for the creation and analysis of pathway-based expression profiles – Supplementay Materials –

Francesco Napolitano[1,2], Diego Carrella[1], Xin Gao[2], Diego Di Bernardo[1,†]

August 31, 2019

[1]Telethon Institute of Genetics and Medicine (TIGEM), Via Campi Flegrei 34, 80078 Pozzuoli (NA), Italy.
[2]Computational Bioscience Research Center, King Abdullah University of Science and Technology (KAUST), Thuwal, 23955-6900, Kingdom of Saudi Arabia.
[†]To whom correspondence should be addressed.

1 Gep2pep, DSEA and Gene2drug

The DSEA [1] and the Gene2drug [2] methodologies are analytical tools based on a pathway-centric approach to gene expression profiles. They were released as closed online webtools[1], and both relied on the MSigDB v4 [3] as gene set collections and Connectivity Map 2.0 [4] as gene expression profiles.

We developed the Gene2drug R/Bioconductor package to allow researchers to perform offline DSEA-like and Gene2drug-like analyses on custom gene expression profiles and custom gene set collections. With Gep2pep it is now possible to apply both kinds of analysis to any set of transcriptional profiles, not necessarily related with drug-induced response, and to any set of pathways, not necessarily related to a target gene of interest. For this reason, within the package terminology, we redefined the "Drug-set Enrichment Analysis" more generically as "Condition-set Enrichment Analysis" (CondSEA) and "Gene2Drug" as "Pathway-set Enrichment Analysis" (PathSEA).

The Gep2pep R/Bioconductor package includes both data management and analysis features. It uses the *Repo* [5] format to internally store and manage expression and gene set data. Parallelization support together with data management allows to perform the conversion process efficiently even on very large datasets. Thus we took advantage of this novel resource to update our previous tools and release new pathway-based data from much larger collections (see Section 3).

2 Features

Gep2pep R/Bioconductor package features include, but are not limited to:

[1]DSEA: http://www.dsea.tigem.it, Gene2drug: http://www.gene2drug.tigem.it

- Conversion of Gene Expression Profiles (GEPs) to Pathway-based Expression Profiles (PEPs).
- Condition-set Enrichment Analysis, AKA DSEA.
- Pathway-set Enrichment Analysis, AKA Gene2drug.
- Direct import from MSigDB gene set collections in XML format.
- Export results to XLS format.
- Parallel computations of GSEAs.
- Seamless, optional support of HDF5 format to handle very large gene set and profile collections.
- Raw mode. In raw mode, converted pathways are not saved directly to the repository. This allows to use parallelization with RHDF5, which does not support concurrent writing. The profiles can be imported at the end of the computation using the dedicated function.
- Bulk creation of merged profiles, obtained according to a merging scheme by averaging enrichment scores and using the Fisher method to aggregate p-values.
- Single Gene Sets, supporting the analysis of gene-based profiles skipping the creation and evaluation of pseudo-sets containing a single gene.
- Multiple consistency checks, such as the existence of all the combinations of profile versus gene-set GSEAs.
- Building pathway-sets for input to gene2drug analysis starting from a logic combination of genes, for example "pathways containing gene X OR Y", "pathways containing gene X AND Y", etc.
- Categorized collections, allowing the use of custom category names for R/Bioconductor BroadCollection objects (otherwise limited to Gene Ontology categories).

3 Updated data and tools released with the package

We took advantage of the Gep2pep package and its parallalization support to update previous pathway-based collections and produce new ones as detailed in this Section. Moreover, we developed a novel version of the DSEA website named DSEA-LINCS (see Subsection 3.3).

3.1 Updated CMap pathway-based profiles

The DSEA and Gene2drug webtools were based on a pathway-based version of the CMap 2.0 and MSigDB 4.0, respectively including 1,309 drugs (after merging profiles corresponding to the same drug, see [1]) and 9,847 gene sets from 10 gene set collections. We released such data in plain text format on the DSEA website. With this paper, on the other hand, we release a novel pathway-based version of the CMap profiles using MSigDB v6.1, which includes 14,645 gene sets from 16 gene set collections. We release such data in Gep2pep format through the new DSEA-LINCS website (see Subsection 3.3).

3.2 New pathway-based LINCS data profiles

The Connectivity Map database has been superseded by a newer collection of drug-induced gene expression profiles within the LINCS [6] project. This allowed us to develop a new version of the DSEA website, named DSEA-LINCS, including a collection of profiles that is larger than the one supported by the current DSEA website by orders of magnitude. The latest release[2] of the LINCS dataset (*Phase II*) consists of $\sim 350,000$ profiles including ~1,500 small molecules assayed in 42 different cellular context at different dosages.

By using a computer cluster and taking advantage of the parallelization support by the Gep2pep package, we were able to convert all the LINCS profiles to pathway-based profiles basing on MSigDB v6.1 (see previous subsection). Since LINCS data include a much more diverse set of cell lines, we included both profiles merged across different cell lines and profiles merged across drug dosages only. This amounted to computing ~350,000,000 Gene Set Enrichment Analyses [7]. Although the methodology behind DSEA-LINCS was previously presented and validated for the DSEA case [1], as a sanity check for the new pathway-based LINCS profiles we measured their ability to predict ATC codes[3] by the World Health Organization, a validation method that we and others previously used in the context of gene expression profiles [8, 9]. Fig. 3.2 shows the results. The database of pathway-based LINCS profiles is freely available for download from the DSEA-LINCS website in Gep2pep format.

3.3 New DSEA-LINCS website

Basing on LINCS pathway-based profiles, we developed a novel version of the DSEA website. Here, together with the Gep2pep package, we thus present a beta version of the DSEA based on LINCS data, which we refer to as DSEA-LINCS[4] in this document. DSEA-LINCS uses Gep2pep as its computational backend and the same data used by the website is available for download for offline use with the R package. See Figure 3.1 for a screenshot showing a usage example.

4 Gep2pep usage

The Gep2pep package is extensively documented, with a vignette and a reference manual available at the Bioconductor website[5]. However, we report here the most fundamental usage steps in order to illustrate its practical application.

4.1 General approach

In order to illustrate the two fundamental steps in any Gep2pep analysis, here we show them generically in the form of pseudo-code. In particular, Algorithm 1 illustrates the necessary procedure to build a working local repository including gene set collections and its use to convert Gene Expression Profiles (GEPs) to Pathway-based Expression Profiles (PEPs). Algorithm 2 shows how, given an existing repository obtained after the execution of Algorithm 1, CondSEA and PathSEA analyses can be easily performed.

[2] https://www.ncbi.nlm.nih.gov/geo/query/acc.cgi?acc=GSE70138
[3] https://www.whocc.no/atc/structure_and_principles
[4] http://dsea.tigem.it/lincs
[5] https://bioconductor.org/packages/release/bioc/html/gep2pep.html

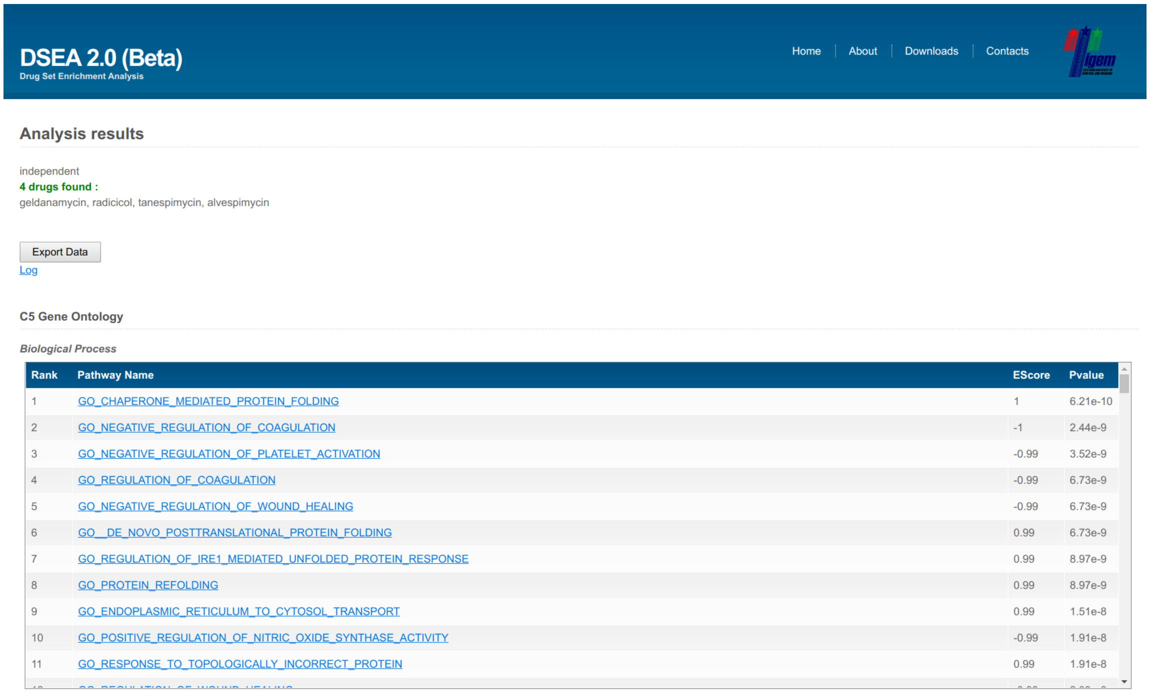

Figure 3.1: DSEA-LINCS website using LINCS data converted and analyzed through the Gep2pep package. The screenshot shows an analysis example including 4 HSP90 inhibitor drugs that we were able to identify in the LINCS database: geldanamycin, radicicol, tanespimycin, alvespimycin. The single most significant pathway found was the very relevant *Chaperone mediated protein folding*.

Algorithm 1 Creation of a local repository of pathway-based expression profiles (PEPs) from gene expression profiles and gene set collections.

Conversion of GEPs to PEPs
input:
    file containing gene set collections G, such as the MSigDB XML file.
    a matrix M where each column is a gene expression profile
output:
    pathway_based conversion of M according to G

1. rep ← Create_Local_Repository(G)
2. ConvertGEPsToPEPs(repo, M)

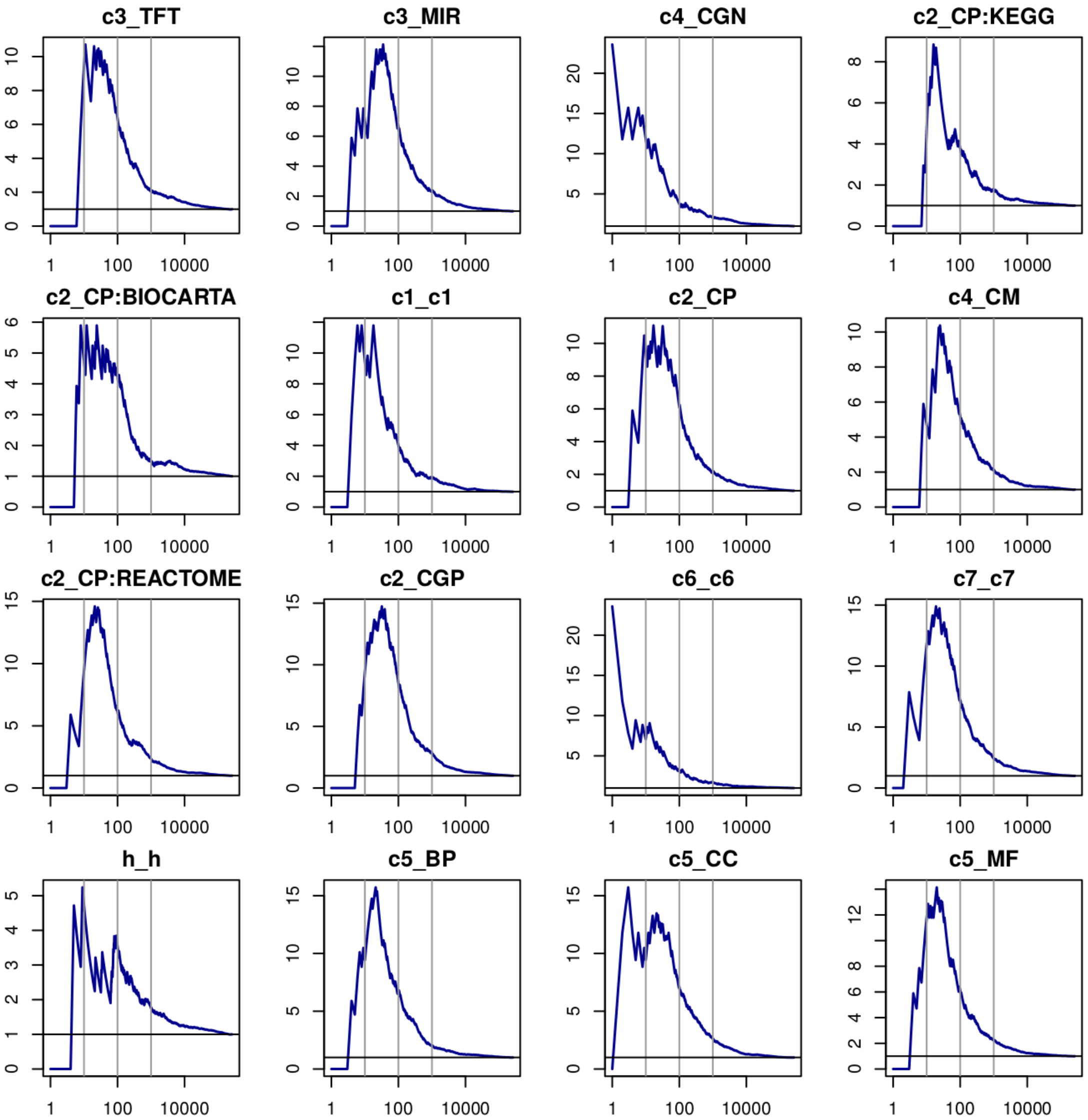

Figure 3.2: Validation of the LINCS profiles after pathway-based conversion. For each pathway collection, all pathway-based profile pairs were compared using the Manhattan distance after ranking by Enrichment Scores. Each subplot shows the validation for a single pathway collection, reporting MSigDB names. All ATC-annotated drug pairs were sorted by similarity and reported on the x axis in log scale (ATC codes were found for 723 LINCS drugs by matching the names, for a total of 261,003 pairs). Positive Predictive Values (PPV) against ATC codes are reported on the y axis after normalization against expected random values. Vertical lines highlight the values of $x = 10$, $x = 100$, and $x = 1,000$. The horizontal line highlights the expected normalized PPV obtained by chance ($y = 1$). For all the pathway collections, the PPV tends to by significantly higher than random when drug pairs are similar (left part of the subplots) and tends to randomness when the drugs are not similar (right part of the subplots).

Algorithm 2 Execution of PathSEA and CondSEA analyses with an existing repository

CondSEA and PathSEA analyses
input:
  path P to an existing repository R of PEPs
  a set C of conditions for the CondSEA, $C \in R$
  a set G of gene sets for the PathSEA, $G \in R$
output:
  CondSEA results
  PathSEA results

1. rep ← Open_Existing_Repository(P)
2. CondSEAout ← CondSEA(rep, C)
3. PathSEAout ← PathSEA(rep, G)

4.2 Working example

Finally we illustrate a working example of CondSEA and PathSEA analyses on real transcriptomic data. In particular, we will use Connectivity Map 2.0 data and the MSigDB v6.1. This would first need the application of Algorithm 1 on such data, which however we performed in advance and made available, as previously described (see Subsection 3.1). The following code downloads, decompress and loads precomputed PEPs for the Connectivity Map together with gene set collection data from the MSigDB v6.1, all in Gep2pep format:

```
> library(gep2pep)                        ## load Gep2pep
> download.file(                          ## download a Gep2pep repository
+              "http://dsea.tigem.it/data/Cmap_MSigDB_v6.1_PEPs.tar.gz",
+              "Cmap_MSigDB_v6.1_PEPs.tar.gz"
+             )
> untar("Cmap_MSigDB_v6.1_PEPs.tar.gz")    ## decompress downloaded repository
> rep <- openRepository("Cmap_MSigDB_v6.1_PEPs")      ## open local repository
```

Now the variable rep contains a handle to the newly downloaded repository of gene sets and PEPs. In the rest of the Section we will perform CondSEA and PathSEA analysis using such repository.

4.3 CondSEA

The code below runs a CondSEA analysis on a set of HSP90 inhibitors using the Gene Ontology pathway collections. This drugs are known to affect the cellular response to stress by acting as chaperons assisting protein refolding.

```
> csea <- CondSEA(                       ## perform CondSEA
+                rep,
+                   c("geldanamycin", "monorden",
+                     "tanespimycin", "alvespimycin"),
+                collections=c("C5_BP", "C5_MF", "C5_CC")
+               )
```

```
> nrow(csea$CondSEA$C5_BP)               ## count rows of results matrix

[1] 4436
```

The last displayed number is the size of the used version of the Biological Process category collection of the Gene Ontology. The following code shows some of the results obtained, including translation of pathway IDs to extended names:

```
> csea$CondSEA$C5_BP[1:3,]               ## show top 3 pathways

        ES         PV
M15275  0.9670498  2.929441e-06
M13557  0.9616858  5.194060e-06
M15562  0.9616858  5.194060e-06

> setId2setName(                         ## convert pathway IDs to names
+     loadCollection(rep, "C5_BP"),
+     rownames(csea$CondSEA$C5_BP)[c(3,14)]
+     )

[1] "GO_PROTEIN_REFOLDING" "GO_PROTEIN_FOLDING"
```

Out of 4,436 gene sets, the pathways "protein folding" and "protein refolding" where ranked in 3rd and 14th positions respectively.

PathSEA

The code below runs a PathSEA on a set of pathways that the GPT gene is involved in. The set is defined by using the *gene2pathways* function. Along the lines of our publication about Gene2drug [2], which used older data, we use only the REACTOME gene sets collection.

```
> pwset <- gene2pathways(rep, "GPT")     ## define the set of pathways
> psea <- PathSEA(                       ## perform PathSEA
+                 rep,
+                 pwset,
+                 collections=c("C2_CP:REACTOME")
+                )
```

The following code shows the top 5 results obtained:

```
> csea$PathSEA$"C2_CP:REACTOME"[1:5,]
              ES          PV
fulvestrant   0.9791667 0.001058196
citalopram   -0.9761905 0.001349200
clonidine    -0.9702381 0.002037028
tomatidine    0.9702381 0.002037028
nifuroxazide  0.9672619 0.002433852
```

We previously demonstrated that both fulvestrant and tomatidine are able to upregulate the expression of the GPT gene [2].

References

[1] Francesco Napolitano, Francesco Sirci, Diego Carrella, and Diego di Bernardo. Drug-Set Enrichment Analysis: A Novel Tool to Investigate Drug Mode of Action. *Bioinformatics*, 32(2):235–241, 2016.

[2] Francesco Napolitano, Diego Carrella, Barbara Mandriani, Sandra Pisonero-Vaquero, Francesco Sirci, Diego L Medina, Nicola Brunetti-Pierri, and Diego di Bernardo. gene2drug: a computational tool for pathway-based rational drug repositioning. *Bioinformatics*, 34(9):1498–1505, dec 2018.

[3] Arthur Liberzon, Aravind Subramanian, Reid Pinchback, Helga Thorvaldsdóttir, Pablo Tamayo, and Jill P. Mesirov. Molecular signatures database (MSigDB) 3.0. *Bioinformatics*, 27(12):1739–1740, jun 2011.

[4] Justin Lamb, Emily D. Crawford, David Peck, Joshua W. Modell, Irene C. Blat, Matthew J. Wrobel, Jim Lerner, Jean-Philippe Brunet, Aravind Subramanian, Kenneth N. Ross, Michael Reich, Haley Hieronymus, Guo Wei, Scott A. Armstrong, Stephen J. Haggarty, Paul A. Clemons, Ru Wei, Steven A. Carr, Eric S. Lander, and Todd R. Golub. The Connectivity Map: Using Gene-Expression Signatures to Connect Small Molecules, Genes, and Disease. *Science*, 313(5795):1929–1935, sep 2006.

[5] Francesco Napolitano. repo: an R package for data-centered management of bioinformatic pipelines. *BMC Bioinformatics*, 18(1):112, dec 2017.

[6] Aravind Subramanian, Rajiv Narayan, Steven M Corsello, David D Peck, Ted E Natoli, Xiaodong Lu, Joshua Gould, John F Davis, Andrew A Tubelli, Jacob K Asiedu, David L Lahr, Jodi E Hirschman, Zihan Liu, Melanie Donahue, Bina Julian, Mariya Khan, David Wadden, Ian C Smith, Daniel Lam, Arthur Liberzon, Courtney Toder, Mukta Bagul, Marek Orzechowski, Oana M Enache, Federica Piccioni, Sarah A Johnson, Nicholas J Lyons, Alice H Berger, Alykhan F Shamji, Angela N Brooks, Anita Vrcic, Corey Flynn, Jacqueline Rosains, David Y Takeda, Roger Hu, Desiree Davison, Justin Lamb, Kristin Ardlie, Larson Hogstrom, Peyton Greenside, Nathanael S Gray, Paul A Clemons, Serena Silver, Xiaoyun Wu, Wen-Ning Zhao, Willis Read-Button, Xiaohua Wu, Stephen J Haggarty, Lucienne V Ronco, Jesse S Boehm, Stuart L Schreiber, John G Doench, Joshua A Bittker, David E Root, Bang Wong, and Todd R Golub. A Next Generation Connectivity Map: L1000 Platform and the First 1,000,000 Profiles. *Cell*, 171(6):1437–1452.e17, nov 2017.

[7] Aravind Subramanian, Pablo Tamayo, Vamsi K. Mootha, Sayan Mukherjee, Benjamin L. Ebert, Michael A. Gillette, Amanda Paulovich, Scott L. Pomeroy, Todd R. Golub, Eric S. Lander, and Jill P. Mesirov. Gene set enrichment analysis: A knowledge-based approach for interpreting genome-wide expression profiles. *Proceedings of the National Academy of Sciences*, 102(43):15545–15550, oct 2005.

[8] Murat Iskar, Monica Campillos, Michael Kuhn, Lars Juhl Jensen, Vera van Noort, and Peer Bork. Drug-Induced Regulation of Target Expression. *PLoS Computational Biology*, 6(9):e1000925, sep 2010.

[9] Francesco Napolitano, Yan Zhao, Vânia M. Moreira, Roberto Tagliaferri, Juha Kere, Mauro D'Amato, and Dario Greco. Drug repositioning: A machine-learning approach through data integration. *Journal of Cheminformatics*, 5(6), 2013.
